# PeMNet for Pectoral Muscle Segmentation

**DOI:** 10.3390/biology11010134

**Published:** 2022-01-14

**Authors:** Xiang Yu, Shui-Hua Wang, Juan Manuel Górriz, Xian-Wei Jiang, David S. Guttery, Yu-Dong Zhang

**Affiliations:** 1 School of Computing and Mathematical Sciences, University of Leicester, Leicester LE1 7RH, UK; xy144@le.ac.uk (X.Y.); shuihuawang@ieee.org (S.-H.W.); 2 Department of Signal Theory, Networking and Communications, University of Granada, 52005 Granada, Spain; gorriz@ugr.es; 3 Department of Computer Science, Nanjing Normal University of Special Education, No.1 Shennong Road, Nanjing 210038, China; 4 Leicester Cancer Research Centre, University of Leicester, Leicester LE2 7LX, UK; 5 Guangxi Key Laboratory of Trusted Software, Guilin University of Electronic Technology, Guilin 541004, China

**Keywords:** pectoral segmentation, deep learning, global channel attention module

## Abstract

**Simple Summary:**

Deep learning has become a popular technique in modern computer-aided (CAD) systems. In breast cancer CAD systems, breast pectoral segmentation is an important procedure to remove unwanted pectoral muscle in the images. In recent decades, there are numerous studies aiming at developing efficient and accurate methods for pectoral muscle segmentation. However, some methods heavily rely on manually crafted features that can easily lead to segmentation failure. Moreover, deep learning-based methods are still suffering from poor performance at high computational costs. Therefore, we propose a novel deep learning segmentation framework to provide fast and accurate pectoral muscle segmentation result. In the proposed framework, the novel network architecture enables more useful information to be used and therefore improve the segmentation results. The experimental results using two public datasets validated the effectiveness of the proposed network.

**Abstract:**

As an important imaging modality, mammography is considered to be the global gold standard for early detection of breast cancer. Computer-Aided (CAD) systems have played a crucial role in facilitating quicker diagnostic procedures, which otherwise could take weeks if only radiologists were involved. In some of these CAD systems, breast pectoral segmentation is required for breast region partition from breast pectoral muscle for specific analysis tasks. Therefore, accurate and efficient breast pectoral muscle segmentation frameworks are in high demand. Here, we proposed a novel deep learning framework, which we code-named PeMNet, for breast pectoral muscle segmentation in mammography images. In the proposed PeMNet, we integrated a novel attention module called the Global Channel Attention Module (GCAM), which can effectively improve the segmentation performance of Deeplabv3+ using minimal parameter overheads. In GCAM, channel attention maps (CAMs) are first extracted by concatenating feature maps after paralleled global average pooling and global maximum pooling operation. CAMs are then refined and scaled up by multi-layer perceptron (MLP) for elementwise multiplication with CAMs in next feature level. By iteratively repeating this procedure, the global CAMs (GCAMs) are then formed and multiplied elementwise with final feature maps to lead to final segmentation. By doing so, CAMs in early stages of a deep convolution network can be effectively passed on to later stages of the network and therefore leads to better information usage. The experiments on a merged dataset derived from two datasets, INbreast and OPTIMAM, showed that PeMNet greatly outperformed state-of-the-art methods by achieving an IoU of 97.46%, global pixel accuracy of 99.48%, Dice similarity coefficient of 96.30%, and Jaccard of 93.33%, respectively.

## 1. Introduction

Breast cancer is one of the most common female cancers worldwide and the second leading cause of mortality in women [1]. Breast cancer commonly affects women between the ages of 35 and 55 and men aged over 40 and over 150,000 deaths are recorded per year [2,3]. In the US, the breast cancer incidence rate has increased slightly per year from 2012 to 2016; however, fatalities declined [4]. Moreover, the contribution of newly developed therapies on reducing mortality rate, breast mammography, a gold standard in the community, has also significantly improved survival due to earlier detection and is of great significance. While there are numerous modalities for breast imaging, mammography is considered to be one of the most effective methods given the feasibility and performance.

The advancement of technology transformed mammography procedures from radiography-based films form to digital form, which was known as full-field digital mammography (FFDM). The advantage of digital mammography is that radiologists are able to magnify mammograms or change the brightness or contrast of mammograms for better interpretation. Another reason digital mammography has gained in popularity is that it is cheap, while acquired images can be stored as Digital Imaging and Communications in Medicine (DICOM) files. Usually, a breast is imaged in two projection planes including Cranio-Caudal (CC) and Medio-Lateral-Oblique (MLO) and in two sides, which leads to LCC, RCC, LMLO, and RMLO, equaling four images. The mammography images are often inspected by a specialist towards identification of abnormalities and localization. However, the complexity of breast tissue and subtlety of cancer in early stages are intrinsic challenges in interpreting mammograms, which itself is a time-consuming task. As radiologists have to read many mammograms in a single day, it is likely that they may fail to show consistent performance when making diagnoses and considering artificial factors such as fatigue or distraction. Studies have shown that at least 10% of cancers are missed during examination [5]. One straightforward way to solve this is to have a second radiologist for interpretation; however, two further problems emerge. One is the inconsistent diagnostic conclusions from the different radiologists. A third radiologist can be invited when there is disagreement on the diagnostic conclusion. However, another problem that needs to be considered is the extra costs of a second read. Instead, computer-aided systems (CADs) for breast cancer analysis have emerged as an attractive alternative. These systems aim to automatically locate and classify abnormalities in mammograms so that radiologists are able to improve their efficiency. Regarding the analysis tasks, CAD systems can be broadly classified into computer-aided detection (CADe), which is mainly responsible for breast abnormality detection (such as breast mass and calcification) and computer-aided diagnosis (CADx) systems that focus on classifying the detected abnormalities or entire images into one of several categories. These two systems can be integrated to form an end-to-end system for higher efficiency, but they can also be separated for specific applications.

Before the prevalence of deep convolutional neural networks (CNNs)-based CAD systems, mammography-based CAD systems for breast cancer analysis mainly consisted of four steps including pre-processing, segmentation, feature extraction and analysis. Pre-processing, which is a crucial step before analysis as the quality of input images possibly determines the bottleneck of subsequent modules, enhances the desired features in the images while depresses the unwanted natures. Segmentation, which plays a key role in image analysis, remains a challenging task while considerable efforts using traditional methods such as threshold methods and active contours-based methods have been made [6]. After segmentation, meaningful features, such as edges and shapes, are extracted by feature extraction and then used for final diagnosis. With the development of deep learning, segmentation, feature extraction and classification can be simply integrated into one single deep learning model. Pre-processing, however, remains too large a topic to be included in single models. For breast cancer analysis, pre-processing mainly includes image enhancement and breast region segmentation. Image enhancement, especially for medical images, is generally applied to improve the brightness, contrast, saturation of images. Given that the size of a mammography image can be thousands by thousands of pixels, breast region segmentation will benefit CAD systems by narrowing down the regions that should be focused on while the efficiency of those systems can be improved as smaller numbers of pixels are involved in computation. The pectoral muscle, which is commonly shown in MLO viewed mammograms, is usually removed before analysis as it can be easily misclassified as fibroglandular tissues. Additionally, artefacts that are accidentally produced during image acquisition may show in pectoral muscle areas of mammography images. Moreover, pectoral muscle regions can be examined by radiologists for auxiliary lymph abnormalities. Aimed at developing a robust and highly efficient breast pectoral muscle segmentation system, we developed an automatic segmentation framework named PeMNet in this paper. Inspired by the work [7,8], we further explored the possibility of combining channel attention architecture with segmentation frameworks. In this study, the datasets used for method evaluation were INbreast and OPTIMAM while segmentation framework is Deeplabv3+ [9,10,11]. The main contributions of this study can be concluded as follows:

- We developed a novel deep learning framework, i.e., PeMNet, that outperformed the performance of the state-of-the-art methods for breast pectoral muscles segmentation in mammograms; Based on the Deeplabv3+ framework, we incorporated deep learning models with the novel attention module and found Incepresnetv2-based segmentation framework performed best among all models. Additionally, the Incepresnetv2-based segmentation framework, which is called $Pe_{IRv2}$ for short, outperformed the state-of-the-art methods by a large margin, showed the IoU of 97.46%, global pixel accuracy of 99.48%, Dice similarity coefficient of 96.30%, Jaccard of 93.33%, respectively, on a merged dataset.
- We proposed a novel attention module named GCAM to extract channel information globally in deep CNNs. Compared to the attention module proposed in [7,8], the proposed attention module is more parameter efficient as fewer learnable training parameters are introduced. By doing so, the number of parameters are then significantly reduced. Furthermore, the proposed attention module can be flexibly integrated with different deep CNN models.
- The proposed attention module is effective for improvement of performance of segmentation frameworks and is of high robustness. At a low parameter-cost, the proposed attention module can greatly improve the performance of the Deeplabv3+ model. Furthermore, this is the first attempt to integrate a novel attention module into any breast pectoral segmentation framework. The experiments on a merged dataset from INbreast and OPTIMAM, where images are collected by different imaging devices, showed the robustness of the proposed model as our model provided consistent segmentation results on the testing set.

This paper is organized as follows. In Section 2, we will briefly review the related works and potential improvements in the area. We then introduce our proposed framework in Section 3 in details, followed by Section 4, where we will introduce more details about the datasets and experimental settings. In Section 5, we will discuss some issues related to the proposed framework and we conclude this paper in Section 6.

## 2. Related Works

Segmentation, a consistently challenging task in the community of computer vision, has also greatly benefited from the development of deep CNNs as semantic segmentation is no longer an exclusive task by human beings. In terms of pectoral segmentation, there have been considerable endeavours towards effective methods aiming at breast pectoral segmentation during recent decades. Before the deep learning era, pectoral segmentation was mainly implemented through following methods including intensity-based methods, region growing methods, line estimation methods, curve estimation methods etc. [2]. In [12], Shrivastava et al. developed a sliding window based algorithm for pectoral muscle removal. In the proposed method, the pectoral muscle is first ensured to be located in the top left region of the wall. A 5 × 5 window was defined to slide over the mammogram while the absolute intensity differences of pixels in the top-left and bottom-right corners of the window are computed. The proposed method achieved 91.3% visual inspection accuracy using the MIAS dataset. Region growing is another widely used technique to estimate pectoral muscle boundaries based on the intensity variations in mammograms. In region growing-based methods, a single seed point inside the pectoral region is selected while pixels that are similar to the seed points are then included in the segmentation results. The segmentation finishes when no more pixels can be included [13]. In another region growing-based method [14], image intensity is rescaled from 0 to 1 while a classical image contrast enhancement method called CLAHE was used to improve the image contrast. The images were then binarized into binary images using a threshold value of 0.03. A set of geometric rules and a region growing method was applied to refine the initial pectoral muscle region. The evaluation of the proposed method on MIAS and DDSM datasets showed promising segmentation results of 95% and 94%, respectively.

Line estimation methods are the most intuitive methods and remain one of the most widely used approaches. A Canny edge detector for pectoral muscle removal was proposed in [15]. In the proposed method, the initial pectoral region was estimated based on a Canny edge detector and the region intensity while the boundary of the pectoral muscle was estimated by straight-line estimation method for refinement. In another similar work [16], a straight-line estimation method was proposed by Zhou et al. for pectoral muscle segmentation. Initial pectoral muscle boundaries were estimated by introducing a Sobel operator for horizontal edges detection while Linear Hough Transform (LHT) was followed to determine the final pectoral muscle boundary. However, no statistical performance measures were given but visual inspection of the accuracy of the segmentation was given. Compared to line estimation methods, curve estimation methods can be considered to be an advanced version of line estimation methods. In the work [17], a cascaded framework for pectoral segmentation was proposed. In the first stage, a four-class K-means clustering method was carried out to cluster the breast pixels into one of the four classes. Then the cluster with highest intensity was taken as the candidate region of pectoral muscle regarding the desired pectoral muscle location. Secondly, the cluster boundary was smoothed by deploying morphological operation, followed by a Hough transform method for initial pectoral muscle boundary extraction. Finally, a second-degree polynomial curve fitting method was applied to initial boundary to obtain the final boundary. Another curve estimation method was proposed in [18], where a multilevel thresholding approach that can successfully segment 96.81% images from MIAS dataset. Based on the assumption that pectoral region could be roughly denoted as an relatively brighter triangle region, initial pectoral muscle region was acquired via a morphological selection algorithm. A cubic polynomial fitting method was then introduced to refine the initial boundary. However, all mentioned methods suffer from several issues. One is that these methods strictly rely on certain restrictions such as the location of pectoral muscle has to be located on the left side of the images. The second one is that some methods are just concluded based on visual segmentation results while no statistical results can be given [15,17]. Given these factors, the robustness and generality of these methods remain to be explored.

Benefitting from facilitating advancement of deep learning, the segmentation task has experienced significant changes as well. For breast pectoral segmentation, there are also some deep learning-based methods [19,20,21,22]. In [20], U-Net was trained on a merged dataset that had 633 MLO view mammograms in the first stage. The region identified with high confidence in the first stage was then refined by a generative adversarial network (GAN) to form the overall pectoral muscle shape. The reported performance of the proposed method outperformed the trained U-Net by 5.1% and 1.9% in Dice similarity coefficient on two datasets, respectively. In another work [21], Ali et al. introduced residual connection into the deep learning model for breast pectoral segmentation. Ten-fold cross-validation on three public datasets including MIAS, INbreast and DDSM showed a mean Intersection over Union (IoU) of 97%, dice similarity coefficient (DSC) of 96% and accuracy of 98%. As can be seen, the above mentioned deep learning-based methods seem to become popular given the advantages on performance. However, developing segmentation frameworks with higher performance still needs more input. In this study, we proposed a novel segmentation framework for pectoral muscles that achieved even higher performance than the state-of-the-art methods by introducing novel attention framework. Furthermore, the proposed method showed higher robustness as consistent segmentation results were found on two datasets with different imaging patterns.

## 3. Methodology

The entire segmentation system can be divided into two components including pre-processing and segmentation components. In the pre-processing module, we will briefly introduce breast region acquisition and view classification as the only basic digital image pre-processing operations involved. In mammography images, breast regions only appear in a limited area and therefore acquisition of breast-only regions at the beginning will significantly reduce the overall computational costs. View classification is also an indispensable module in an automatic breast cancer analysis system so that MLO view mammograms can be segmented accordingly while CC view mammograms can be analyzed directly. In the segmentation module, we will briefly revisit the architecture of the segmentation framework Deeplabv3+. Then we will introduce the details of the proposed attention model GCAM. We then conclude the segmentation section with the overall structure of the proposed segmentation framework PeMNet.

### 3.1. Pre-Processing

**Figure 1:**
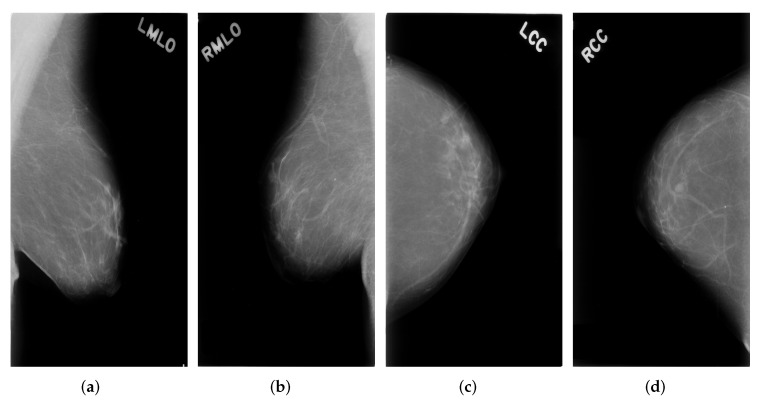
Four example mammograms of one breast (**a**) Left MLO view images (**b**) Right MLO view images (**c**) Left CC view images (**d**) Right CC view images.

One breast usually has four mammograms including LMLO, RMLO, LCC, RCC. An example can be seen in Figure 1.

**Figure 2:**
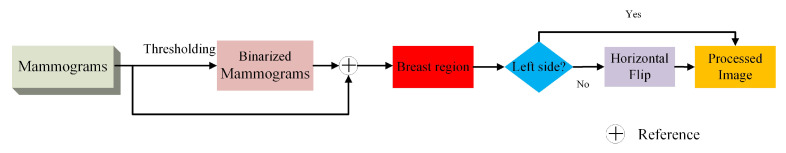
Data flow in pre-processing module. Reference means the beast image is acquired from original mammograms by referring to the binarized mammograms.

The purpose of pre-processing is to align the pectoral wall to the left side of the image and then extract the breast region for the following modules. The data flow of our proposed pre-processing framework can be seen in Figure 2.

**Figure 3:**
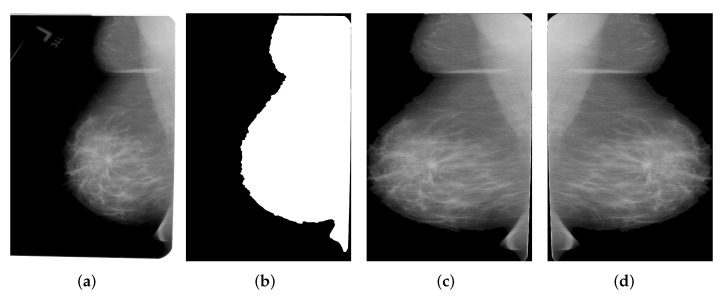
Breast region extraction (**a**) Original mammogram (**b**) Binarized mammogram (**c**) Extracted breast region (**d**) Flipped breast region.

In Figure 2, the pectoral wall is on the left hand side of the mammogram when the number of non-zero pixel values on the left side outnumbers the number of non-zero pixel values on the right hand side. Otherwise, the pectoral wall, which is on the right hand side of a mammogram, is flipped with the mammogram to the left hand side. Furthermore, as can be seen from Figure 1a, the real breast region only occupies a small area of the entire mammogram and should be extracted to avoid unnecessary computational cost. To do this, we chose 20 as the threshold value to binarize the images and the biggest connected components are then taken as the masks for the breasts. Morphological opening operation is applied to remove disturbing objects such as characters or artefacts produced during image acquisition process. Regarding the masks, we are able to extract breast regions from the whole mammogram. The resultant images corresponding to each procedure can be seen in Figure 3.

The extracted breast region images in the first stage of pre-processing are then classified into MLO view and CC view based on GoogLeNet. In this study, we used transfer learning technique for view classification as we used GoogLeNet trained on a natural image classification tasks as the source network instead of training it from scratch. The reason we used GoogLeNet is that mammography views are quite different from each other so no over-large models should be applied. Considering this, we used GoogLeNet as the backbone, as it is a relative small scale network with decent performance on image classification tasks [23]. To adapt GoogLeNet for our view classification task here, we simply removed the top layers including the classification layer in the original GoogLeNet and added two new fully connected layers and a dropout layer, and then fine-tuned the newly generated network on our dataset for view classification.

### 3.2. Segmentation Module

#### 3.2.1. Revisit Deeplabv3+

**Figure 4:**
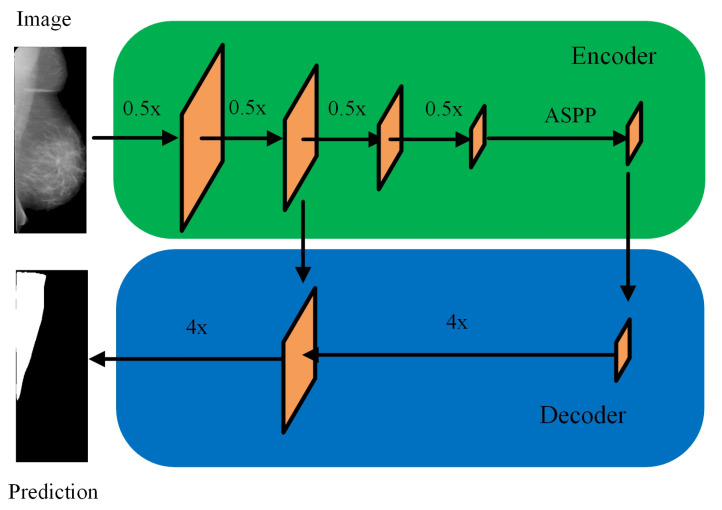
Encoder-Decoder with dilated convolution in Deeplabv3+.

Compared to Unet and SegNet [24,25], Deeplabv3+ model has shown to be preferable given its performance [26,27]. As a result, in this study, we took Deeplabv3+ as the segmentation framework. Deeplabv3+ deploys an encoder-decoder structure that can simultaneously encode multi-scale contextual information and capture the boundaries of sharper objects when recovering the spatial information via decoder. The novelty of Deeplabv3+ is that depthwise separable convolution is embedded into the Atrous Spatial Pyramid Pooling (ASPP) and decoders module, where ASPP is the improved version of SPP with Atrous convolution or dilated convolution. The introduction of depthwise separable convolution and dilated convolution is to reduce the parameters of the framework while the performance of the framework will not be harmed. The encoder-decoder architecture is shown in Figure 4. One more advantage of Deeplabv3+ is the flexibility of combination with different deep CNN models. Therefore, we chose ResNet18, ResNet50, MobileNetv2, XceptionNet and InceptionResNetv2 [28,29,30] as the backbones for Deeplabv3+ in this study. All these models are state-of-the-art deep CNN models that achieved high accuracy on image classification challenge and have been widely used in computer vision tasks such as detection, regression besides classification.

#### 3.2.2. Global Channel Attention Module

Attention mechanism, which allows humans to focus on salient areas instead of processing the whole scene, plays an important role in human visual task [31]. To improve the performance of deep learning models, experts in the community have explored possible methods to integrate attention mechanism into those models [32,33]. In this study, we aimed at extracting global channel attention maps for segmentation performance improvement, we, therefore, proposed to embed a light-weighted attention module titled GCAM into our PeMNet framework [7].

**Figure 5:**
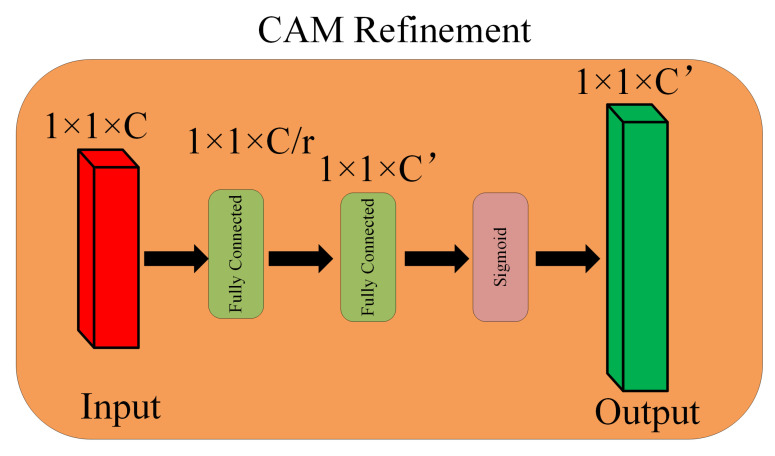
CAM refinement module.

Given an image **I** $\in R^{H\times W\times 3}$ and the intermediate feature maps after certain convolutional blocks in deep CNNs as $M^{x}\in R^{W_{x}\times H_{x}\times C_{x}}$ in feature level *x*, where *W*, *H* stands for width, height of the image **I**, respectively. $W_{x}$, $H_{x}$ and $C_{x}$ stands for the width, height and number of channels of $M^{x}$, respectively. In GCAM, global max-pooling and global average-pooling are deployed to obtain 1D CAMs from the feature maps in certain depth of deep CNNs, which can be denoted as:

(1) $$M_{cat}^{x}=\left[GMP\left(M_{x}\right);GAP\left(M_{x}\right)\right]$$

where $M_{cat}^{x}$ in $R^{1\times 1\times 2C_{x}}$ is the concatenated CAMs, [·] means concatenation operation, GMP(·) and GAP(·) stands for global maximum pooling and global average pooling, respectively. $M_{cat}^{x}$ refined by CAM refinement module to produce $M_{RCAMs}^{x}$ in $R^{1\times 1\times 2C_{x+1}}$, which refers to refined CAM here. $C_{x+1}$ is the number of channel map in feature level x+1. The detailed architecture of the refinement module can be seen in Figure 5.

The refinement process can be defined as:

(2) $$M_{RCAMs}^{x}=MLP\left(M_{cat}^{x}\right)=\delta \left(W_{1}\delta \left(W_{2}M_{cat}^{x}\right)\right)$$

where $W_{1}$ and $W_{2}$ stands for the weights of hidden layer and output layer, respectively. To reduce the number of parameters in the MLP, a shrinking rate *r* is normally introduced. By doing so, the total number of parameters becomes

(3) $$\frac{C}{r}\times C+C^{{}^{\prime }}\times \frac{C}{r}$$

i.e.,

(4) $$\frac{C}{r}\left(C+C^{{}^{\prime }}\right)$$

where *C* is the number of input channel while $C^{{}^{\prime }}$ stands for the number of output channel.

**Figure 6:**
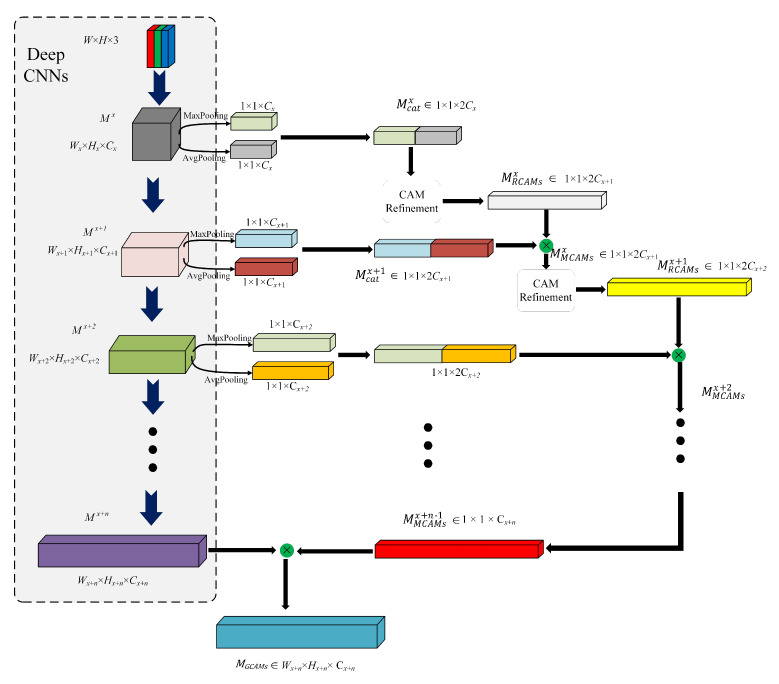
Global channel attention module.

The acquired $M_{RCAMs}^{x}$ are then multiplied with concatenated CAMs in next feature level of deep CNNs, as can be expressed as:

(5) $$M_{MCAMs}^{x+1}=M_{RCAMs}^{x}\cdot M_{Cat}^{x+1}$$

where $M_{MCAMs}^{x+1}$ in $R^{1\times 1\times 2C_{x+1}}$ stands for resultant CAM after the multiplication of $M_{RCAMs}^{x}$ and $M_{Cat}^{x+1}$. · indicates elementwise multiplication here. Similarly, $M_{MCAMs}^{x+1}$ is then refined by the CAM refinement module to produce the refined CAMs $M_{RCAMs}^{x+1}$ for next feature level. By repeating these procedures for multiple times, we then have the final $M_{MCAMs}^{x+n-1}$, which is then multiplied with final feature maps directly without further CAM refinement and results in the refined feature maps $M_{GCAMs}$, which is

(6) $$M_{GCAMs}=M^{x+n}\cdot M_{MCAMs}^{x+n-1}$$

where $M^{x+n}$ stands for the feature maps at feature level x+n. The Detailed procedures of GCAM can be seen in Figure 6.

#### 3.2.3. Overall Segmentation Architecture

**Figure 7:**
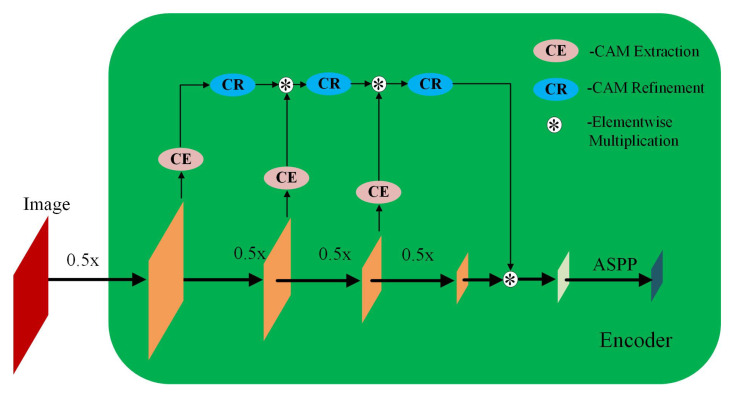
The architecture encoder in PeMNet.

The final feature map $M_{GCAMs}$ is then forwarded to Atrous Spatial Pyramid Pooling (ASPP) module in the framework of Deeplabv3+ for feature resampling prior to convolution. The refined encoder in the proposed PeMNet can be seen in Figure 7. Note that the architecture of decoder is relatively simple than that of encoder so that we keep it unchanged in the proposed model. By doing so, we then have our proposed PeMNet.

**Figure 8:**
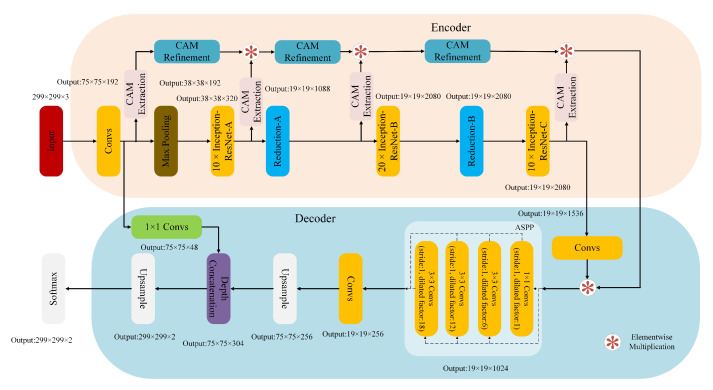
The architecture of PeMNet with the backbone of InceptionResNetv2.

A detailed architecture of our segmentation model $PeMNet_{InceptionResNetv2}$ that takes InceptionResNetv2 as the backbone can be seen in Figure 8.

In $PeMNet_{InceptionResNetv2}$, the “Convs” indicates the stem of InceptionResNetv2 that produces feature maps of 0.25 height and width of the input images.

## 4. Experiment

In this section, we will begin with the details of the datasets used in this study. Then, we will move to the measurements for performance evaluation of the view classification and segmentation, followed by pectoral segmentation results. Finally, we will compare our proposed framework with the state-of-the-art methods to show the advantages of our proposed framework.

### 4.1. Experiment Configurations

The segmentation model was trained on the SPECTRE High-Performance Computing Facility at the University of Leicester with a single GPU Tesla P100 PCI-E(16GB). The training parameters are listed in Table 1 when training the segmentation model on the merged dataset. When training the model, devices with large GPU memory are recommended as the training time can be greatly reduced by increasing the minibatch size. Here, we just fixed the minibatch size to be 32 to avoid possible memory leaks when training large models such as Deeplabv3+s that uses InceptionResNetv2 as the backbone.

### 4.2. Dataset

In this study, we used two datasets, namely OPTIMAM and INbreast, to evaluate the performance of the proposed framework. In total, we merged 682 MLO view mammography images from the OPTIMAM dataset and 200 MLO view mammography images from the INbreast dataset as the new dataset. We then randomly chose 80% (545 images) from the OPTIMAM dataset and 80% (160 images) from INbreast for training while the remaining 20% of each dataset were used for evaluation. Detailed composition of the training set and testing set can be seen in Table 2.

**Figure 9:**
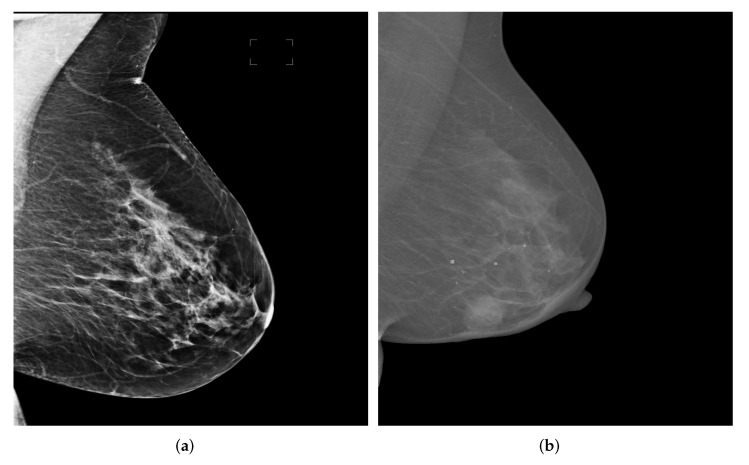
Mammography image examples from OPTIMAM and INbreast datasets. (**a**) An example image from OPTIMAM. (**b**) An example image from INBreast.

In Figure 9, we show two examples from the two datasets for intuitive interpretation. As can be seen, breast regions only appear in the top left corner while there is a large margin on the right hand size of the images. Therefore, the pre-processing procedure is meaningful to extract breast-only regions from the images and reduces potential overall computational cost. As for the image contrast between pectoral muscle and breast region, the mammography image from OPTIMAM dataset has better contrast as it shows a salient boundary between pectoral muscle and breast while the pixel intensities of the pectoral muscle area and breast area in images from INBreast seem to be more homogeneous.

### 4.3. Measurements

For segmentation, we used $P_{GT}$ to stand for the area of true pectoral muscle while $P_{P}$ stands for the area of predicted pectoral muscle. The number of predicted pectoral pixels that are true pectoral pixels are denoted as *TP* while the correctly predicted non-pectoral pixels are denoted as *TN*. *FP* stands for number of pixels that are wrongly segmented as the pectoral muscle while *FN* stands for number of pectoral muscle pixels that is segmented as background. Based on these values, we are able to measure the segmentation performance from Intersection of Union (*IoU*), Global Pixel Accuracy (*GPA*), Dice Similarity Coefficient (*DSC*), Jaccard coefficient, Sensitivity and Specificity. The definition of *IoU* is given in Equation (7) as:

(7) $$IoU=\frac{|P_{GT}\cap P_{P}|}{|P_{GT}\left|+\right|P_{P}\left|-\right|P_{GT}\cap P_{P}|}$$

*GPA* is expressed as:

(8) $$GPA=\frac{TP+TN}{TP+TN+FP+FN}$$

Similarly, *DSC* can be written as:

(9) $$DSC=\frac{2|P_{GT}\cap P_{P}|}{|P_{GT}\left|+\right|P_{P}|}$$

*Jaccard* coefficient can be calculated through:

(10) $$Jaccard=\frac{|P_{GT}\cap P_{P}|}{|P_{GT}\cup P_{P}|}$$

Sensitivity and Specificity, which are two common metrics for classification task evaluation, are introduced here to evaluate the performance of segmentation models on segmenting true pectoral muscle and true background. The reasons why we include these two metrics are mainly two fold. One is that the values of sensitivity and specificity determines the values of *IoU* and *GPA*, which mean these two metrics are indispensable metrics. Another reason is that we can have a more intuitive understanding of the model on segmenting the true pectoral muscle area, which is indicated by sensitivity. The definitions are given below.

(11) $$Sensitivity=\frac{TP}{TP+FN}$$

(12) $$Specificity=\frac{TN}{TN+FP}$$

### 4.4. Pectoral Segmentation Results

As mentioned before, we deployed numerous deep CNNs for the segmentation task in this study. Before we embed GCAM into our PeMNet, we first trained and tested the performance of the original Deeplabv3+ models based on them. We repeatedly trained the models ten times and then have ten individual models evaluated on the test set. The results on the test set are given below in Table 3. $Deeplabv3{+}_{ResNet18}$, which is $DL_{ResNet18}$ for short, means the Deeplabv3+ model that takes ResNet18 as the backbone and so forth. For better comparison, we also compared the performance of Unet with Deeplabv3+ [24]. Correspondingly, the number of learnable training parameters are shown in Table 4.

**Figure 10:**
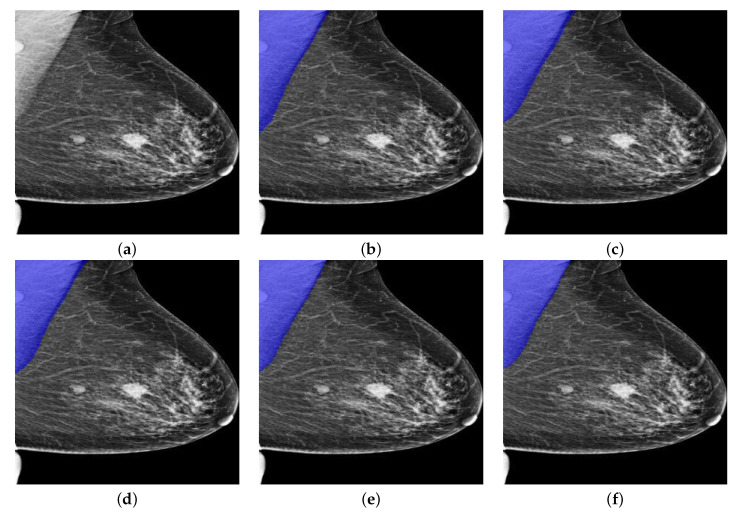
A segmentation example from OPTIMAM by Deeplabv3+ with different backbones. (**a**) Pre-processed image. (**b**) DLResNet18: 98.29% of IoU, 99.73% of GPA, 97.70% of DSC, 95.50% of Jaccard, 99.89% of Sensitivity, and 99.72% of Specificity. (**c**) $DL_{ResNet50}$: 99.18% of IoU, 99.87% of GPA, 98.35% of DSC, 96.75% of Jaccard, 99.90% of Sensitivity, and 99.87% of Specificity. (**d**) $DL_{MobileNetv2}$: **99.35**% of IoU, **99.90**% of GPA, **98.64**% of DSC, 97.32% of Jaccard, **100**% of Sensitivity, and 99.89% of Specificity. (**e**) $DL_{XceptionNet}$: 98.87% of IoU, 99.83% of GPA, 98.08% of DSC, 96.23% of Jaccard, 98.04% of Sensitivity, and 99.99% of Specificity. (**f**) $DL_{InceptionResNetv2}$: 98.99% of IoU, 99.85% of GPA, 98.30% of DSC, 96.65% of Jaccard, 98.15% of Sensitivity, and **100**% of Specificity.

As can be seen in Table 3, all Deeplabv3+ models showed over 95% of IoU, 99% of GPA, 95% of Sensitivity and Specificity, which validated the effectiveness of deeplabv3+ model for pectoral segmentation task. However, the overall DSC and Jaccard metrics remained to be low as the averaged DSC is just around 95% while the averaged Jaccard is only around 90%. Nevertheless, the model based on InceptionResNetv2 consistently showed high performance in terms of IoU, GPA, DSC, Jaccard and Specificity though the Sensitivity is slightly lower than other models. As can be seen from Table 4, the Deeplabv3+ model that takes InceptionResNetv2 as backbone showed predominating performance due to the depth of InceptionResNetv2 and the number of the training parameters. Interestingly, the Deeplabv3+ model that takes MobileNetv2 as the backbone showed much higher performance than UNet. This finding further boosted our choice on using Deeplabv3+ as the basic framework. One segmentation example from OPTIMAM by Deeplabv3+ with different backbones is given in Figure 10. The blue areas in the figures indicate the segmentation results given by the segmentation models.

**Figure 11:**
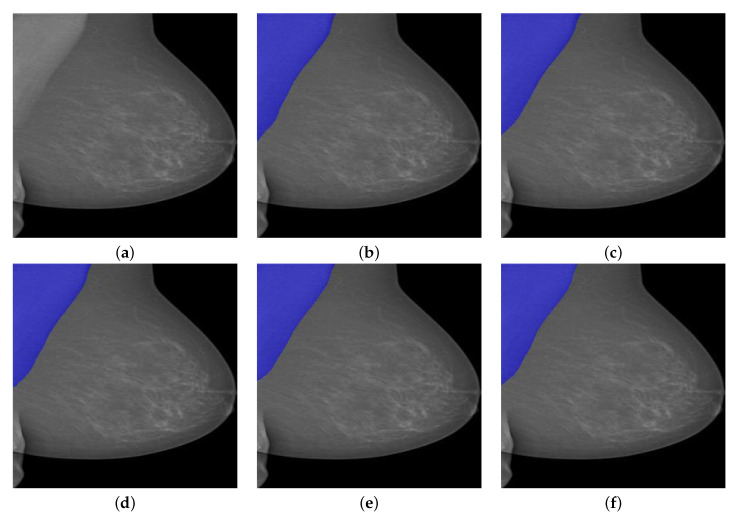
A segmentation example from INbreast by Deeplabv3+ with different backbones. (**a**) Pre-processed image. (**b**) $DL_{ResNet18}$: 97.99% of IoU, 99.80% of GPA, 96.85% of DSC, 93.89% of Jaccard, **100**% of Sensitivity, and 99.60% of Specificity. (**c**) $DL_{ResNet50}$: 98.60% of IoU, 99.86% of GPA, 97.39% of DSC, 94.92% of Jaccard, **100**% of Sensitivity, and 99.72% of Specificity. (**d**) $DL_{MobileNetv2}$: 98.15% of IoU, 99.82% of GPA, 96.82% of DSC, 93.83% of Jaccard, **100**% of Sensitivity, and 99.63% of Specificity. (**e**) $DL_{XceptionNet}$: 98.87% of IoU, 99.83% of GPA, 98.08% of DSC, 96.23% of Jaccard, 98.04% of Sensitivity, and **99.99**% of Specificity. (**f**) $DL_{InceptionResNetv2}$: **99.21**% of IoU, **99.88**% of GPA, **98.28**% of DSC, **96.62**% of Jaccard, 99.89% of Sensitivity, and 99.86% of Specificity.

As can be seen from Figure 10a, there are two masses in the breast region while there is one more mass-like artefact in the pectoral muscle. In this scenario, pectoral segmentation plays a key role in removing the artefact, which turns out the be the side benefit of pectoral removal. The segmentation results seem to quite similar while the MobileNetv2-based model seems to give the best results as it consistently provides highest IoU, GPA, DSC and Sensitivity. Another segmentation example from INbreast dataset by Deeplabv3+ with different backbones is shown in Figure 11.

We can see from Figure 11a that the lower part of the pectoral muscle has a very weak boundary between it and the breast region, which could be a challenging situation for traditional image segmentation methods. However, all Deeplabv3+ models successfully segmented the pectoral muscle while the InceptionResNetv2-based one performed best among all models in terms of all evaluation metrics except Sensitivity.

We then tested the performance of the proposed PeMNet on the test set while the segmentation results on the test set can be seen in Table 5. $Pe_{R18}$, $Pe_{R50}$, $Pe_{Mov2}$, $Pe_{Xcep}$, $Pe_{IRv2}$ stands for PeMNet that takes ResNet18, ResNet50, MobileNetv2, XceptionNet and InceptionResNetv2 as the backbones, respectively. Similarly, we compared the number of training parameters of different models in Table 6, where the last column indicates the number of the increased parameters of PeMNet compared to Deeplabv3+ models.

**Figure 12:**
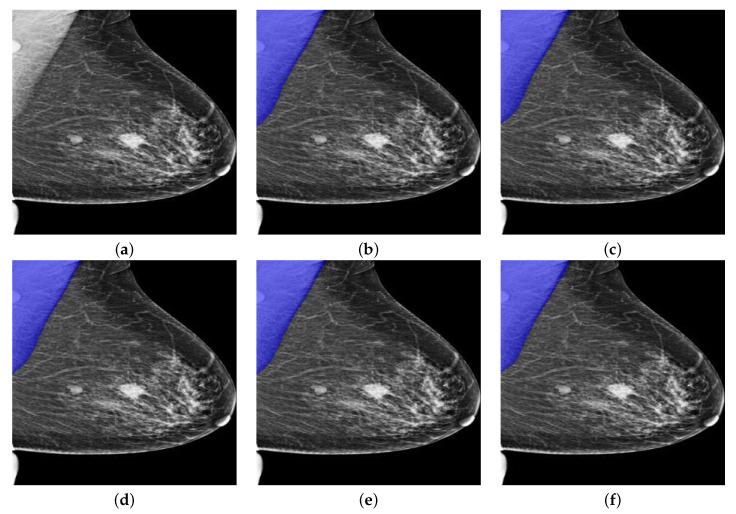
A segmentation example from OPTIMAM by PeMNet with different backbones. (**a**) Pre-processed image. (**b**) $Pe_{R18}$: 99.00% of IoU, 99.70% of GPA, 98.41% of DSC, 96.87% of Jaccard, 99.53% of Sensitivity, and 99.88% of Specificity. (**c**) $Pe_{R50}$: 99.53% of IoU, 99.92% of GPA, 99.03% of DSC, 98.08% of Jaccard, **99.90**% of Sensitivity, and 99.93% of Specificity. (**d**) $Pe_{Mov2}$: **99.57**% of IoU, **99.78**% of GPA, **99.20**% of DSC, **98.41**% of Jaccard, 99.59% of Sensitivity, and **99.97**% of Specificity. (**e**) $Pe_{Xcep}$: 99.43% of IoU, 99.72% of GPA, 99.00% of DSC, 98.01% of Jaccard, 99.49% of Sensitivity, and 99.95% of Specificity. (**f**) $Pe_{Inv2}$: 99.49% of IoU, 99.76% of GPA, 99.03% of DSC, 98.08% of Jaccard, 99.56% of Sensitivity, and 99.95% of Specificity.

As can be seen from Table 5, $Pe_{IRv2}$ performed best among all PeMNets. Moreover, $Pe_{IRv2}$ beats the best-performing Deeplabv3+ model, i.e., $Deeplabv3{+}_{InceptionResNetv2}$, by a significant margin as $Pe_{IRv2}$ achieved much higher evaluation metrics. Furthermore, the parameter increment of different PeMNets showed a linear relationship with the depth of backbones, where $Pe_{IRv2}$ again gained the highest increment. However, as can be seen from Table 3 and Table 5, the performance of some PeMNets is even worse than the counterpart models. The reason behind this could be the depths of these models are much shallower for meaningful CAMs to be extracted and therefore be used when compared to PeMNet based on InceptionResNetv2. The segmentation example from OPTIMAM by PeMNet can be found in Figure 12.

Same as basic Deeplabv3+ models, all PeMNets achieved successful segmentations but with better performance in terms of IoU, DSC and Jaccard. Visually, the segmentation results are quite similar to each other. In this case, however, $Pe_{Mov2}$ obtained the highest values from IoU, GPA, DSC, Jaccard and Specificity and therefore is considered the best-performing model.

**Figure 13:**
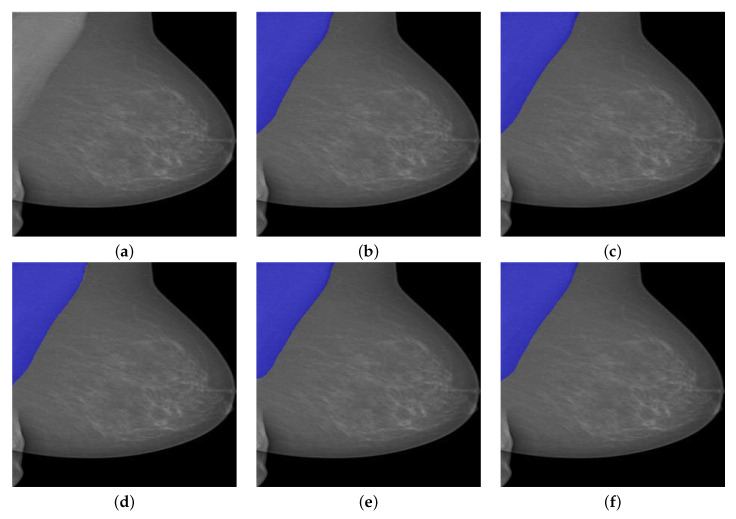
A segmentation example from INbreast by PeMNet with different backbones. (**a**) Pre-processed image. (**b**) $Pe_{R18}$: 99.27% of IoU, 99.93% of GPA, 98.40% of DSC, 96.85% of Jaccard, **100**% of Sensitivity, and 99.86% of Specificity. (**c**) $Pe_{R50}$: 99.42% of IoU, 99.94% of GPA, 98.55% of DSC, 97.14% of Jaccard, **100**% of Sensitivity, and 99.89% of Specificity. (**d**) $Pe_{Mov2}$: **99.46**% of IoU, 99.84% of GPA, 98.67% of DSC, 97.37% of Jaccard, 99.75% of Sensitivity, and 99.92% of Specificity. (**e**) $Pe_{Xcep}$: 99.64% of IoU, **99.96**% of GPA, 98.85% of DSC, 97.72% of Jaccard, **100.00**% of Sensitivity, and 99.93% of Specificity. (**f**) $Pe_{IRv2}$: **99.91**% of IoU, **99.96**% of GPA, **99.14**% of DSC, **98.30**% of Jaccard, 99.96% of Sensitivity, and **99.95**% of Specificity.

Similarly, we then performed our trained PeMNets to the same example image from INbreast for comparison. The results are shown in Figure 13.

As can be seen from Figure 13, all PeMNets presented successful segmentation results while $Pe_{IRv2}$ provided best segmentation results with 99.91% of IoU, 99.96% of GPA, 99.14% of DSC, 98.30% of Jaccard, 99.96% of Sensitivity, and 99.95% for Specificity. Furthermore, it is worth noting that some PeMNets, such as $Pe_{R50}$, $Pe_{Xcep}$, also achieved comparable segmentation results while some of them even obtained 100% Sensitivity. From the above experiments, we can conclude that $Pe_{IRv2}$ was the best model for pectoral muscle segmentation in terms of the evaluation metrics. However, $Pe_{Mov2}$ turned out to be preferable considering the trade-off between the size of the model and the performance gained.

**Figure 14:**
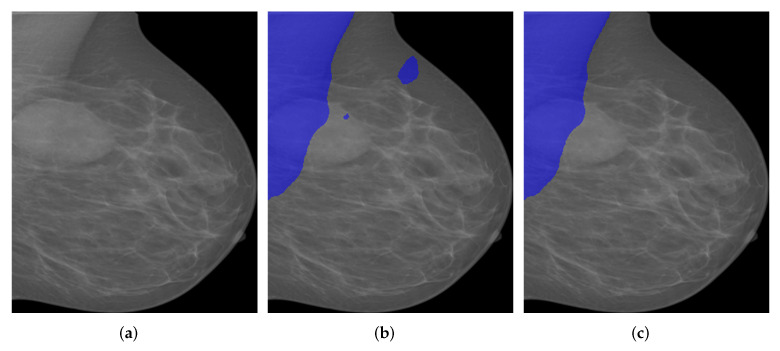
A segmentation example by $Pe_{IRv2}$ when tumor is adjoining to pectoral muscle. (**a**) Pre-processed image. (**b**) Original segmentation results by $Pe_{IRv2}$. (**c**) Post processed segmentation results.

However, mammography images can be complicated where breast tumors may even be adjoining or close to pectoral muscles though it is quite rare. One example can be seen in Figure 14. We then segmented the image via the proposed PeMNet and the result is shown in Figure 14a. Post-processing, the segmentation results are refined to be more precise as shown in Figure 14b.

As can be seen, $Pe_{IRv2}$ successfully segmented the real pectoral muscle from breast tissue and the tumor. Instead of relying on context information in the images for segmentation, $Pe_{IRv2}$ effectively followed a semantic segmentation pattern. The situation when breast tumors are located in the pectoral muscle is also quite rare and can be quite obvious to be distinguished from common mammography images.

**Figure 15:**
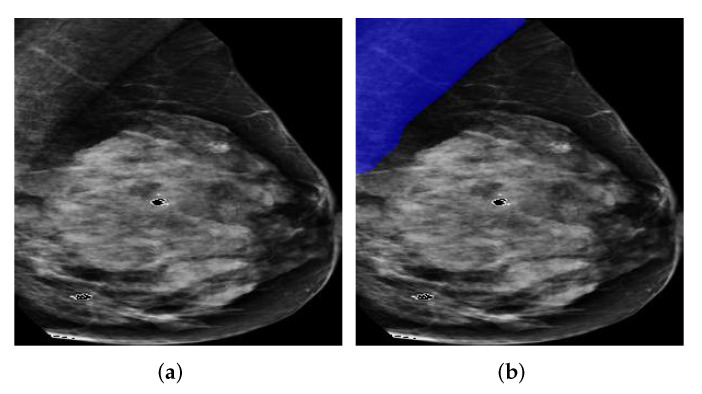
A segmentation example by $Pe_{IRv2}$ when image is of low contrast. (**a**) A low contrast mammography image. (**b**) Segmentation results by $Pe_{IRv2}$.

**Figure 16:**
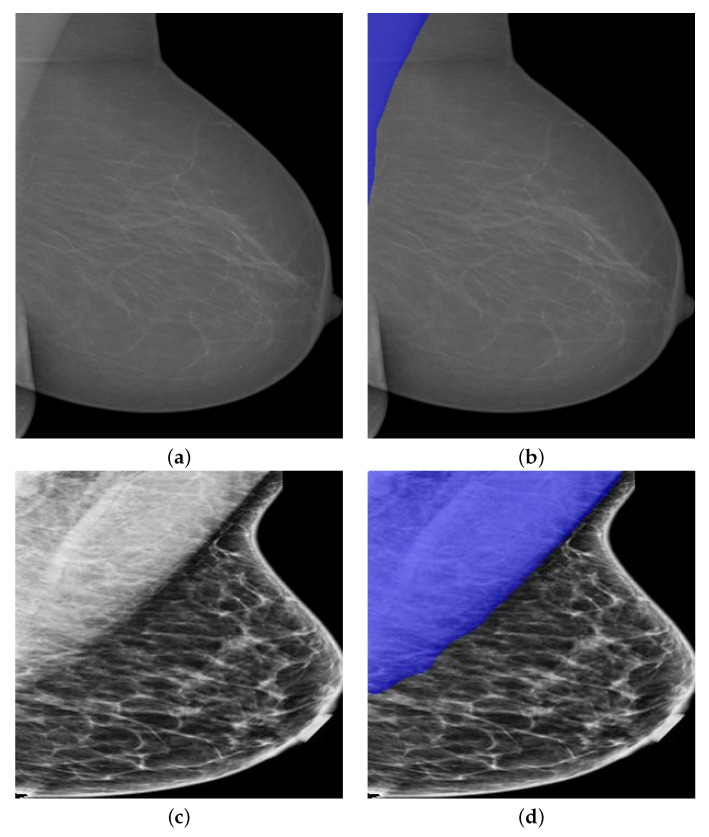
Segmentation examples by $Pe_{IRv2}$ with varied size of pectoral muscle. (**a**) Mammography images in the presence of the small pectoral muscle. (**b**) Segmentation results by $Pe_{IRv2}$. (**c**) Mammography images in the presence of the large pectoral muscle. (**d**) Segmentation results by $Pe_{IRv2}$.

The variations of the pectoral muscles, such as the low image contrast, too small or too big pectoral muscle areas, can also lead to challenging pectoral segmentation. In Figure 15a, the quality of the image seems to be poor as the upper part of the pectoral muscle is not visually clear from the breast area. However, the segmentation result is quite visually accurate as the pectoral muscle has been correctly segmented out from breast area. More specifically, the lower part of the pectoral muscle seems to be connected to the breast tissues in the image. However, the proposed model successfully partitioned the pixels into pectoral muscle and breast without taking breast tissues as pectoral muscle. The size of pectoral muscle may also vary from mammogram to mammogram and thus post threat to stable and accurate segmentation results. In Figure 16, we showed some possible situations in practice.

In Figure 16a, the real pectoral muscle region is quite small in the mammogram. However, $Pe_{IRv2}$ still correctly segmented the pectoral muscle area though over segmentation is induced slightly. On the contrary, the pectoral muscle region could be quite big in the mammograms under some situations, as shown in Figure 16c. The segmentation results in Figure 16d is of high accuracy as the edge of the segmented pectoral muscle is smooth. From the above experiments, we believe that PeMNet, especially $Pe_{IRv2}$, can be used for pectoral muscle segmentation in MLO-view mammography images.

### 4.5. Method Comparison

In this section, we will compare our proposed segmentation methods with the state-of-the-art methods. The results are presented in Table 7.

As can be seen, our proposed method showed predominating performance compared with the state-of-the-art methods. Additionally, our proposed method has the highest IoU, GPA, DSC, and Sensitivity among all methods.

## 5. Discussion

Given the importance of breast pectoral segmentation, many efforts ranging from traditional methods to the state-of-the art deep CNNs methods have been performed. However, it remains a problem that must be resolved. One main issue concerning breast pectoral segmentation is the lack of large-scale well-annotated datasets for training of high performance models. In recent years, considerable effort has been devoted to developing intelligent and robust methods for breast pectoral segmentation. However, the majority of the methods are evaluated on self-annotated public datasets or even private datasets due to the limited availability of datasets. In this study, we evaluated our segmentation framework both on access limited dataset, i.e., OPTIMAM and on a public dataset named INbreast. Based on Deeplabv3+ model, we integrated the proposed novel attention module into PeMNet for image segmentation task. Compared to traditional methods that suffered from poor performance, our method turned out to be more reliable with higher performance. Compared to the deep CNN based methods, our proposed novel PeMNet still offers the architectural novelty while the performance of our model remains to be the best performing one compared to other methods.

Another issue with the models for pectoral segmentation is the robustness of the methods. Before the advent of deep learning, feature-based methods dominated the field. However, the robustness of these kinds of systems remain to be improved as minor changes in the images could lead to failure of the systems. Therefore, the advantage of deep learning-based methods is such that the robustness has been drastically enhanced. In terms of robustness, the proposed segmentation framework has been proven to be robust against various situations and turned out to be suitable for pectoral muscle segmentation tasks.

## 6. Conclusions

In this study, we successfully developed an automatic breast pectoral segmentation model named PeMNet for mammogram pre-processing in mammography image analysis. The key of the model is the proposed novel attention model that was architecturally friendly to deep CNNs and therefore can be easily repurposed for new computer vision tasks. By integrating the attention module, our proposed PeMNet framework showed highest performance on pectoral muscle segmentation.

Nevertheless, there are still some limitations to this study. One problem is the effectiveness of the proposed attention module remains to be improved. As can be seen from the experiment, the PeMNet with shallow deep CNNs backbones performed even worse than Deeplabv3+ models with same backbones. The reason could be from the dataset perspective as the datasets for validation are still quite small. As we mentioned before, the publicly available datasets for breast pectoral segmentation are quite limited. Therefore, we may validate the proposed attention module on larger-scale datasets in future. However, there is still further work that can be done from the perspective of architecture as further exploration on architecture should be done. Another issue is the choice of backbones for the segmentation model. In this study, we simply deployed numerous deep CNNs as the backbones, but more state-of-the-art models should be explored for better performing segmentation models in future. 

## Figures and Tables

**Table 1:** Training parameters for pectoral segmentation model.

Parameter	Value
Minibatch size	32
Initial learning rate	1 × 10${}^{-4}$
Maximum training epochs	50
Learning rate drop factor	0.5
Learning rate drop period	20
Shuffle per epoch	Yes
Loss function	Cross-entropy

**Table 2:** Dataset composition for pectoral muscle segmentation.

Data	OPTIMAM	INbreast	Total
Training set	545	160	705
Testing set	136	40	176
Total	682	704	881

**Table 3:** Performance of the trained Deeplabv3+ based on different deep CNN models.

Model	IoU	GPA	DSC	Jaccard	Sensitivity	Specificity
Unet	76.09 ± 10.08	92.13 ± 2.91	72.87 ± 10.38	60.77 ± 11.97	98.43 ± 0.43	91.37 ± 6.17
$DL_{ResNet18}$	96.65 ± 0.57	99.30 ± 0.15	94.80 ± 0.82	90.47 ± 1.47	98.44 ± 0.42	99.41 ± 0.18
$DL_{ResNet50}$	96.87 ± 0.75	99.35 ± 0.22	95.21 ± 1.28	91.19 ± 2.24	**98.43** ± **0.66**	99.47 ± 0.26
$DL_{MobileNetv2}$	96.47 ± 0.34	99.27 ± 0.24	94.97 ± 0.57	90.68 ± 1.11	97.33 ± 0.62	99.52 ± 0.15
$DL_{XceptionNet}$	96.65 ± 0.55	99.30 ± 0.25	94.80 ± 0.92	90.52 ± 1.61	98.11 ± 0.62	99.46 ± 0.19
$DL_{InceptionResNetv2}$	**97.13** ± **0.28**	**99.42** ± **0.48**	**95.60** ± **0.40**	**92.29** ± **0.36**	96.64 ± 1.96	**99.77** ± **0.10**

**Table 4:** Number of training parameters of different models.

Model	Number of Layers	Number of Parameters
UNet	46	7,697,410
$DL_{ResNet18}$	100	20,594,356
$DL_{ResNet50}$	206	43,923,380
$DL_{MobileNetv2}$	186	6,749,044
$DL_{XceptionNet}$	205	27,579,844
$DL_{InceptionResNetv2}$	853	71,045,012

**Table 5:** Performance of the trained PeMNets.

Model	IoU	GPA	DSC	Jaccard	Sensitivity	Specificity
$Pe_{R18}$	96.98 ± 0.50	99.38 ± 0.20	95.33 ± 0.84	91.45 ± 1.53	98.28 ± 0.56	99.51 ± 0.18
$Pe_{R50}$	96.78 ± 0.78	99.33 ± 0.21	95.06 ± 1.20	90.93 ± 2.11	98.41 ± 0.56	99.45 ± 0.24
$Pe_{Mov2}$	96.45 ± 0.33	99.27 ± 0.24	94.78 ± 0.45	90.65 ± 0.87	97.43 ± 0.58	99.50 ± 0.13
$Pe_{Xcep}$	96.70 ± 0.48	99.32 ± 0.49	94.93 ± 0.58	90.93 ± 1.12	97.61 ± 1.11	99.54 ± 0.18
$Pe_{IRv2}$	**97.46** ± **0.45**	**99.48** ± **0.29**	**96.30** ± **0.66**	**93.33** ± **1.04**	97.12 ± 0.56	**99.78** ± **0.07**

**Table 6:** Number of training parameters of PeMNets.

Model	Number of Layers	Number of Parameters	Parameters Increment
$Pe_{R18}$	116	20,760,500	166,144
$Pe_{R50}$	220	44,909,876	986,496
$Pe_{Mov2}$	200	6,927,964	178,920
$Pe_{Xcep}$	226	28,787,168	1,207,324
$Pe_{IRv2}$	874	80,670,948	9,625,936

**Table 7:** Method comparison. The bold font indicates the best.

Method	Dataset	IoU	GPA	DSC	Jaccard	Sensitivity	Specificity
Shen et al. [18]	INbreast	-	-	89.10 ± 16.54	84.61 ± 18.15	-	-
Soleimani et al. [19]	INbreast	-	-	95.60 ± 8.40	92.60 ± 10.60	95.20 ± 8.6	**99.80** ± **1.80**
Ali et al. [21]	INbreast	87.9 ± 4.5	95.00 ± 3.15	94.00 ± 3.72	-	-	-
Rampun et al. [34]	INbreast	-	-	89.60 ± 10.10	84.60 ± 15.60	89.60 ± 9.60	99.70 ± 0.80
Guo et al. [20]	Private	-	-	96.22 ± 0.05	-	-	-
$Pe_{IRv2}$	INbreast	**97.46** ± **0.45**	**99.48** ± **0.29**	**96.30** ± **0.66**	**93.33** ± **1.04**	**97.12** ± **0.56**	99.78 ± 0.07
(Our method)	OPTIMAM						

## Data Availability

Not applicable.
